# The Cognitive Profile of Persons with Obsessive Compulsive Disorder with and without Autism Spectrum Disorder

**DOI:** 10.2174/1745017901814010304

**Published:** 2018-11-30

**Authors:** Elizabeth Ekman, Arto J. Hiltunen

**Affiliations:** Department of Social and Psychological Studies, Section of Psychology, Karlstad University, Karlstad, Sweden

**Keywords:** ASD, OCD, Cognitive Profile, RAS, RIQ, Interpretation

## Abstract

**Introduction:**

Autistic Spectrum Disorder (ASD) is often comorbid with Obsessive Compulsive Disorder (OCD). But to what extent can obsessional symptoms in individuals with ASD be considered “genuinely” comorbid OCD – or are there other mechanisms that are related to ASD? Which mechanisms in OCD with and without ASD share common features? People with ASD have a cognitive profile characterized by “mindblindness”; the antecedent is often referred to in terms of not knowing how to perform or behave and this is the cause of discomfort. This raises the question whether individuals with ASD and comorbid OCD share the same cognitive elements of responsibility interpretation and the same fear of causing harm as individuals who merely have OCD.

**Objective:**

The aim of the present study is therefore to evaluate the extent of responsibility interpretation in individuals with OCD alone compared with people experiencing OCD in the context of ASD.

**Methods:**

Two instruments, the Responsibility Attitude Scale (RAS) and the Responsibility Interpretations Questionnaire (RIQ), were administered to three groups of participants: (i) individuals diagnosed with OCD (n = 32); (ii) individuals with ASD and OCD (n = 19); and (iii) non-clinical control participants (n = 23).

**Results:**

Results indicate significant differences in all measures of responsibility belief (interpretation of obsession and assumption of responsibility) between the OCD-only group and the two other groups.

**Conclusion:**

The conclusion is that OCD in people with ASD is not as “genuine” as in people with only OCD, according to cognitive behavioral theory of OCD.

## 1 INTRODUCTION

Patients diagnosed with autism spectrum disorder (ASD) have disproportionately high levels of comorbidity with anxiety disorders in general and obsessive compulsive disorder (OCD) in particular [1-4]. It has been shown that the prevalence of both obsessions and compulsions in persons with ASD is comparable with that in persons with OCD; however, ASD patients may have more compulsions than obsessions and there is evidence that the content of the obsessions in ASD persons is more focused on order and organization, symmetry, and sexuality [2, 3, 5].

A cognitive behavioural theory of OCD holds that the key factor influencing the occurrence and maintenance of OCD is how the intrusive cognition (obsession) is interpreted by the individual in terms of personally relevant threats and the individual’s sense of responsibility for causing and/or preventing harm [6]. Such assumptions, in turn, mediate interpretations such as the concern that the fear of being responsible for harm or its prevention can lead to discomfort and motivation to attempt to prevent, undo or neutralize the obsession [7].

There is evidence that persons with OCD are more likely to hold general assumptions and beliefs about responsibility, as well as related appraisals of intrusions, compared with non-OCD patients. Salkovskis’ model of OCD shows how an early experience and/or a critical life event can activate a general assumption of responsibility for harm. For example, patients with OCD may believe that not preventing a catastrophe is the same thing as causing the catastrophe, which leads to efforts to prevent a foreseen disaster, regardless of how unlikely it may appear – a process that triggers and perpetuates OCD [6, 8].

It has been suggested that, psychologically, the key to understanding OCD lies in understanding the beliefs of people with OCD: the need to be completely certain of preventing harm, primed by the occurrence of intrusions, and seek to ensure that they are not causing harm [6, 9]. Symptoms such as stereotyped, repetitive patterns of behaviour are included in the diagnostic criteria for ASD in The Diagnostic and Statistical Manual of Mental Disorders, fifth edition (DSM-5), as well as for OCD. The two diagnoses share common criteria [10], but in OCD the repetitive behaviour is related to the antecedent, *i.e*. an obsession [1].

Diagnostically, OCD includes both obsessions – that is, recurrent and persistent thoughts, urges or impulses that are experienced as intrusive, disgusting, unseemly or otherwise ego-dystonic, and give rise to fear of causing harm – and compulsions, which are repetitive observable behaviours or covert behaviours typically accompanied by short-term decreases in anxiety, distress and uncertainty [11, 12]. The obsessional “idea” is often considered possible or even likely because the person is having the thought. This in itself arises from the fear that thinking something increases the risk that the incident will occur [11, 13] (so-called “thought–action fusion (TAF)”). Studies have shown that TAF is stronger in patients with OCD than in non-OCD patients [11-12].

From this point of view, it becomes important to recognize the role of the antecedent in maintaining repetitive behaviours in order to understand the differences between the various disorders. The conceptualization of the behavioural and cognitive analysis of the disorders may be one way of showing the differences between them [11].

There has been considerable discussion about whether the phenomenology and topography of OCD symptoms in persons with ASD can be regarded as the same as in other types of OCD. Baron-Cohen and Wheelwright [14] and Bejerot [15] have argued that we have no data on obsessional symptoms in children with ASD in terms of whether or not these are genuine OCD symptoms. There seem to be differences in the symptom content in people with autism compared with other groups, and ASD individuals may differ in the extent to which they are distressed and ego-dystonic. In ASD, obsessions are often described in terms of all-consuming and often repetitive interests, referred to as “special interests”, that are phenomenologically quite different from obsessions occurring in primary OCD [14-16].

It is well established that individuals with ASD have cognitive difficulties in the form of “mindblindness” – also referred to as problems of “theory of mind”. This is defined in terms of difficulties in perceiving and understanding “unspoken” or “invisible”, *i.e*. the implicit meaning, including the non-verbal communication, in a dialogue or in information given [17-19]. Furthermore, the cognitive profile of persons with ASD shows more systemizing tendencies compared with the norm, and ASD is associated with exceptional ability to recognize patterns and see details [20, 21]. This often leads to misunderstandings and uncertainty.

Clinical surveys of OCD in individuals with ASD have found that they often do not have the knowledge of how to perform an act or a behaviour, *i.e*. they do not know how to act in different situations; also, they have flaws in mental imagery and are unable understand how to react, which may lead to discomfort [15]. For example, they might not know how to precisely clean the table or wash their hair or body, which can result in anxiety based on not knowing whether they are “correct”. They might end up with constant compulsive behaviour in order to be sure. Baron-Cohen and Wheelwright [14] likened the obsessions in ASD to “folk physics” – people’s basic knowledge of how the physical world works. This means that the obsessions are questions about and an interest in understanding how things function, in order to understand how to act. Terms such as “physics people” and “maths people” refer to the profile of their cognitive abilities, and folk science (folk psychology) is a commonsensical way of describing the understanding and predicting of the natural and social worlds.

The aim of the present study was to evaluate the extent of responsibility interpretations in individuals with OCD alone relative to people experiencing OCD in the context of ASD [6, 8, 13].

Individuals with OCD and ASD were compared with individuals with OCD but without ASD in terms of general assumptions concerning responsibility and related interpretations of intrusions [6, 8, 12, 13]. To our knowledge there are no previous studies about the content of obsessional thoughts in individuals with OCD and ASD, and the way they interpret intrusions, concerning the possibility of causing harm, or assume responsibility for harm.

## 2 METHODS

### 2.1 Participants

The sample consisted of adult persons, women and men (Table **1**). There were three groups of participants: individuals diagnosed with ASD plus OCD (n = 19), participants with only OCD (n = 32) and a benchmarking group of non-clinical participants (n = 23).

The OCD patients had been diagnosed by an authorized psychologist/psychotherapist at their psychiatric clinic according to standard psychiatric assessment, including an interview, the Yale-Brown obsessive-compulsive (Y-BOCS) symptom checklist, ** the Mini International Neuropsychiatric Interview (MINI), the Attention Deficit Hyperactivity Disorder (ADHD) Self-Report Scale (ASRS-v1.1), the Alcohol Use Disorder Identification Test for consumption (AUDIT-C), the Health Questionnaire (EQ-5D), and the Patient Health Questionnaire (PHQ 9), and diagnosis was based on DSM-4 criteria *.* The diagnoses of clients with ASD had been made by the authorized neuropsychiatric team (consisting of a neuropsychologist, neuropsychiatrist, occupational therapists, physical therapists, special education teachers, speech therapists and counsellors) after a standard neuropsychiatric examination including neuropsychological testing and an interview. In the diagnostic process, any suspicion of multiple diagnoses led to further examination and assessment. As no information was obtained in our sample about presence of multiple diagnoses - except for a few instances that led to exclusion from the study – we were confident that there were no multi-diagnoses in our sample.

Fifty-six individuals were recruited from various psychiatric clinics, treatment centres and patient associations in Karlstad, Arvika, Hofors, Stockholm and Gothenburg. The non-clinical controls consisted of 23 psychology and sport psychology students at Karlstad University, the Swedish School of Sport and Health Sciences (GIH), and Gothenburg University. All participants were engaged in a daily activity, *i.e*. work or school, and all were of normal intelligence or higher. The ASD group were all high-functioning and had no cognitive limitations or difficulties.

### 2.2 Procedure

A cross-sectional design was used in the present study. The students were recruited by teachers who also handed out the instruments and the consent form. All instruments and consent forms were sent back to the researcher at the university in pre- addressed, stamped envelopes. All students could contact their teacher or the researcher if there were questions.

The non-control participants were recruited either by the contact person at the clinic or by the patient association. They were given information about the project, and the opportunity to ask questions. After consent to participate in the study was obtained, the questionnaires were administered by the responsible clinician by mail or in person. The study was carried out in the participants’ homes or at their place of work/daily activities, and the questionnaires were completed by the participants in their own time. Participants sent the consent form and the questionnaires to the researcher by mail in pre-addressed, stamped envelopes.

In all, 79 participants were enrolled in the study, However, four non-controls were excluded for not meeting the criteria (two with bipolar disorder, one with developmental disorder with no OCD diagnosis, and one with psychosis) and a fifth non-control was excluded for being under age.

Participation to the study was voluntary, and the study was approved by the Regional Ethics Review Board in Uppsala (2014/519, revised 2015-04-15).

### 2.3 Instruments

Two instruments were used to assess responsibility beliefs [6]: the Responsibility Attitude Scale (RAS), comprising 26 questions assessing general beliefs about responsibility, and the Responsibility Interpretations Questionnaire (RIQ), containing 22 questions assessing the frequency and conviction to specific interpretations of intrusive thoughts about the possibility of causing damage. In RAS, ratings are on a seven point scale (totally agree, agree very much, agree slightly, neutral, disagree slightly, disagree very much, totally disagree), on what attitude is typical of your way of looking at things most of the time, in terms of feeling responsible for thoughts on causing harm (scoring 0-182).

In RIQ, the respondents are asked whether the itemized thoughts have occurred to them during the past 2 weeks. They also rated to what extent they believed these interpretations, using a visual analogue scale (VAS) from 0 = “I did not believe this idea at all” to 100 = “I was completely convinced that this idea was true.” Examples of intrusive thoughts are: “If I don’t resist these thoughts it means I am being irresponsible”; “I’ll feel awful unless I do something about this thought”; “Since I’ve had this thought I must want it to happen”; “Now I’ve thought of bad things that could go wrong I have a responsibility to make sure I don’t let them happen”; “There’s nothing wrong with letting thoughts like this come and go naturally”; and “This is just a thought so it doesn’t matter.”

The four scores derived from the RIQ are described by Salkovskis *et al*. [6] as an F1 score, which provides a frequency score relating to high-responsibility items and is obtained by calculating the mean for 16 statements in Section F1 on a scale from 0 (“never occurred”) to 4 (“always occurred”); an F2 score, which provides a mean frequency score relating to six low-responsibility items in Section F2; a B1 score, which provides a mean percentage belief score for each of the 16 high-responsibility items ranging from 0% = “I did not believe this idea at all” to 100% = “I was completely convinced this idea was true” in Section B1; and a B2 score, which provides a mean percentage belief score for the six low- responsibility items in Section B2. In this study, however, B1 and B2 scores were calculated on a scale of 0-10 instead of 0-100.

The RAS and RIQ instruments were translated into Swedish and back-translated into English, and have been checked by Professor Paul Salkovskis. Salkovskis *et al*. [6] have reported reasonably good psychometric characteristics for the RAS and RIQ, with Cronbach’s alpha 0.92-0.93.

### 2.4 Data Analysis

Data analysis was carried out to compare general assumptions of responsibility and related interpretations of intrusions between the groups with the primary variable responsibility belief based on the RAS score. Secondary comparisons were made between the groups based on the four scores derived from the RIQ.

The data were analysed using SPSS (SPSS Inc., Chicago, IL, USA). A one-way independent analysis of variance (ANOVA) *F*-test was used to analyse differences between the control, OCD and ASD+OCD groups, and Tukey’s Honest Significant Difference (HSD) test was applied to prevent a mass significance problem in post-hoc comparisons.

## 3 RESULTS

There were no differences in the background variables gender [χ(2) = 2.78, non-significant (NS)] and age [*F*(2.68) = 2.65, NS] between the three groups.

**Fig. (1):**
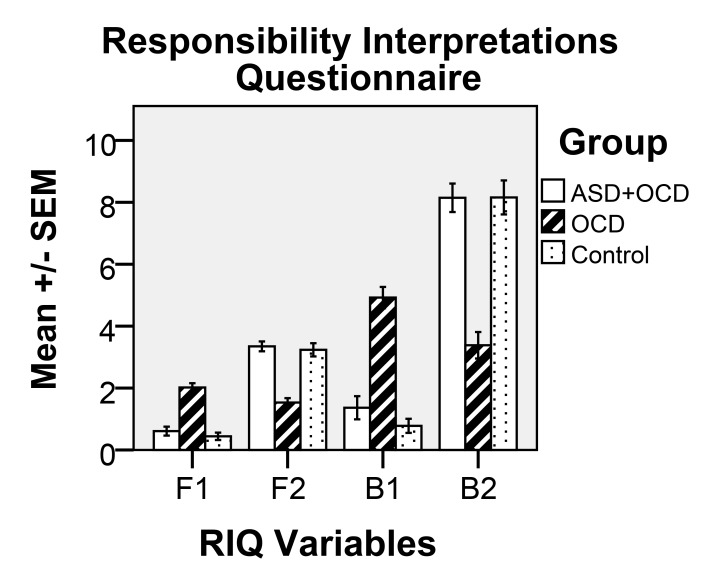
Differences in Responsibility Interpretations Questionnaire (RIQ) variables between the three groups investigated. The RIQ variables on the x-axis are: **F1:** a frequency score relating to high-responsibility items; **F2:** a frequency score relating to low-responsibility items; **B1:** a percentage belief score for each high-responsibility idea; and **B2:** a percentage belief score for each low-responsibility idea. ASD = autism spectrum disorder; OCD = obsessive compulsive disorder; SEM = standard error of mean. **Note**: Values on the y-axis (0-10) concerning B1 and B2 correspond with 0-100%.

**Fig. (2):**
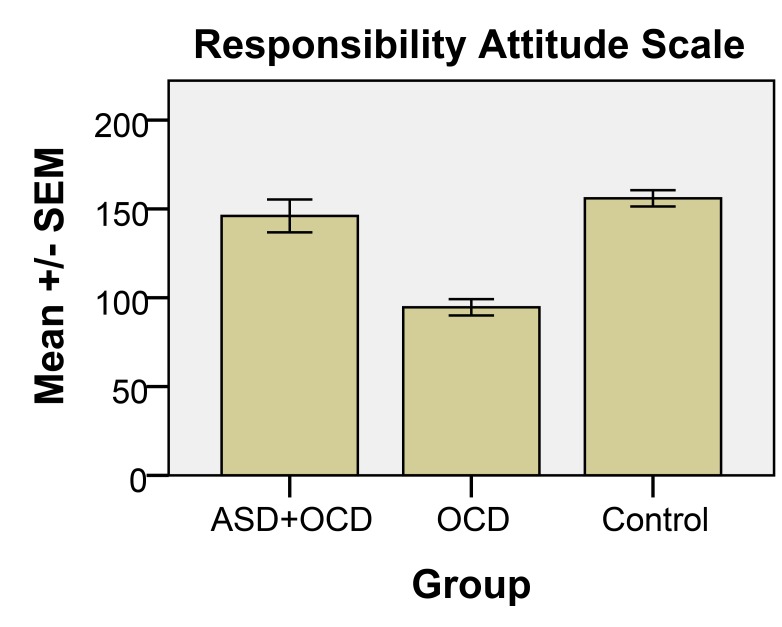
Differences in Responsibility Attitude Scale (RAS) variables between the three groups investigated. ASD = autism spectrum disorder; OCD = obsessive compulsive disorder; SEM = standard error of mean. **Note**: Low scores in RAS indicate high responsibility for the intrusive thought.

One-way ANOVA showed statistically significant differences between the groups for the majority of mean values in the four RIQ scales (F1, F2, B1 and B2, cf. Fig. **1**) and the RAS (cf. Fig. **2**), as follows: F1, F(2, 69) = 42.99, p<0.001; F2, F(2, 68) = 38.91, p<0.001; B1, F(2, 68) = 53.0, p<0.001; B2, F(2, 68) = 36.94, p<0.001; and for RAS: F(2, 67) = 34.97, p<0.001. Post-hoc tests (Tukey’s HSD) showed significant (p = 0.001) differences for all variables (F1, F2, B1, B2 and RAS) between OCD and the two other conditions (OCD+ASD and control).

## 4 DISCUSSION

The main results of the current study show a significant difference between participants with ASD and OCD and participants with only OCD concerning RAS and RIQ scores. There were no significant differences between participants with both ASD and OCD and the non-clinical control group. All non-control participants were attending an established psychiatric clinic and had been diagnosed there, based on a standardized diagnostic system in accordance with Swedish health care policy, and the diagnosis was assumed to have been correct. Our main concern in this study was to differentiate the two groups concerning their responses in the two instruments used, the RAS and RIQ, which, according to Salkovsksis, is an indicator for OCD diagnosis and OCD patients’ problems in terms of interpretation of intrusive thoughts and taking excessive responsibility for their content. Based on the participants’ responses to the RAS and RIQ, it became clear that the OCD group answered in line with expectations for this group, as has also been shown in other studies [22-24]. The responses of the ASD+OCD group were clearly different from the OCD group, and much more like the control group’s responses.

One explanation for the significant results is that individuals with ASD do not interpret intrusions because of their systematic thinking and mindblindness [17, 25]. They are probably more prone to rule-based behaviour as a response to what Baron-Cohen and Wheelwright [14] refer to as “folk physics”. They want to understand how the social and physical worlds work and become anxious when they cannot perform at their best. A relevant question is why not all individuals with ASD develop compulsions that result in anxiety and OCD-like behaviour. Is it related to their environment and upbringing – for example, the help provided by parents and school – or is it due to how individuals with ASD understand the surrounding world? Is it related to where on the autism spectrum the person falls, or is it a combination of all of the above?

There are also individuals who develop social anxiety. Some will engage in extreme avoidance behaviour. To understand these various anxiety problems, it is necessary to look at the comorbidity of the anxiety problems for ASD from a perspective based on the cognitive profile in ASD. Today there is lack of knowledge about and consensus in understanding the various anxiety disorders in individuals with ASD [26].

In our study, the results of the control group probably mean that they have a cognitive profile with no catastrophic interpretations of intrusive thoughts, resulting in RIQ and RAS scores that are the same as for persons with ASD, suggesting they do not believe that their intrusive thoughts can cause harm. Nor do they think they are responsible for the intrusive thoughts. To explain the concept of folk physics and mindblindness, we would like to quote one of the participants with ASD asking, with respect to the RAS questions, “How can I know how and whether I interpret my thoughts if I can’t see my interpretation?”

The participants were asked whether the RIQ and RAS questions were difficult to understand. Many participants with ASD and many members of the control group found the questions difficult to understand. Two of the participants with ASD asked if they could answer the F1 questions “the other way around”. Studies have shown that individuals with OCD almost selectively endorse questions about common OCD symptoms [6]. In this study, when asked whether they found the RIQ and the RAS questions difficult, the participants with OCD answered “No”, often adding “This is how I think.”

There is a great need for further studies on the difference in the cognitive profile in OCD with and without ASD, as well as with ADHD. We also need to learn more about cognitive behavioural therapy (CBT) treatments both for individuals with ASD and OCD and for people with ADHD and OCD, and other anxiety problems they might have, by understanding the differences in their cognitive profiles. When it comes to persons with ASD and OCD, we must fill the gap between lack of ability to form a mental image and imagining the whole picture, including what is invisible in the situation, by using CBT in various different modifications using visualization [27]. Further studies are also needed to investigate persons with ADHD and OCD, which will be another challenge.

## Figures and Tables

**Table 1:** Participants’ demographic data.

**Variable**	**ASD+OCD group** (*n* = 19)	**OCD group** (*n* = 32)	**Control group** (*n* = 23)
**Gender** Female	7 (36.8%)	19 (59.4%)	10 (43.5%)
Male	12 (63.2%)	13 (40.6%)	13 (56.5%)
**Age, yrs** M (±SD)	31.0 (8.92)	30.96 (11.3)	37.48 (12.68)

ASD = Autism Spectrum Disorder; M = Mean; OCD = Obsessive Compulsive disorder; SD = Standard Deviation.
